# Role of extracytoplasmic function sigma factors in biofilm formation of *Porphyromonas gingivalis*

**DOI:** 10.1186/1472-6831-15-4

**Published:** 2015-01-17

**Authors:** Satosu Onozawa, Yuichiro Kikuchi, Kazuko Shibayama, Eitoyo Kokubu, Masaaki Nakayama, Tetsuyoshi Inoue, Keisuke Nakano, Yukinaga Shibata, Naoya Ohara, Koji Nakayama, Kazuyuki Ishihara, Toshiyuki Kawakami, Hiromasa Hasegawa

**Affiliations:** Department of Oral Microbiology, Matsumoto Dental University, 1780 Gobara, Hiro-oka, Shiojiri, Japan; Oral Health Science Center, Tokyo Dental College, 2-9-18, Misaki-cho, Chiyoda-ku, Tokyo, Japan; Department of Microbiology, Tokyo Dental College, 2-1-14, Misaki-cho, Chiyoda-ku, Tokyo, Japan; Department of Oral Microbiology, Graduate School of Medicine, Dentistry and Pharmaceutical Sciences, Okayama University, 2-5-1 Shikata-cho, Kita-ku, Okayama, Japan; Department of Oral Pathology, Matsumoto Dental University School of Dentistry, 1780, Hirooka-Gobara, Shiojiri, Japan; Hard Tissue Pathology Unit, Matsumoto Dental University Graduate School of Oral Medicine, 1780, Hirooka-Gobara, Shiojiri, Japan; Division of Microbiology and Oral Infection, Department of Molecular Microbiology and Immunology, Nagasaki University Graduate School of Biomedical Sciences, Nagasaki, Japan

**Keywords:** Extracytoplasmic function sigma factor, Biofilm, *Porphyromonas gingivalis*, Periodontal disease

## Abstract

**Background:**

*Porphyromonas gingivalis* has been implicated as a major pathogen in the development and progression of chronic periodontitis. *P. gingivalis* biofilm formation in the subgingival crevice plays an important role in the ability of the bacteria to tolerate stress signals outside the cytoplasmic membrane. Some bacteria use a distinct subfamily of sigma factors to regulate their extracytoplasmic functions (the ECF subfamily). The objective of this study was to determine if *P. gingivalis* ECF sigma factors affect *P. gingivalis* biofilm formation.

**Methods:**

To elucidate the role of ECF sigma factors in *P. gingivalis*, chromosomal mutants carrying a disruption of each ECF sigma factor-encoding gene were constructed. Bacterial growth curves were measured by determining the turbidity of bacterial cultures. The quantity of biofilm growing on plates was evaluated by crystal violet staining.

**Results:**

Comparison of the growth curves of wild-type *P. gingivalis* strain 33277 and the ECF mutants indicated that the growth rate of the mutants was slightly lower than that of the wild-type strain. The PGN_0274- and PGN_1740-defective mutants had increased biofilm formation compared with the wild-type (*p* < 0.001); however, the other ECF sigma factor mutants or the complemented strains did not enhance biofilm formation.

**Conclusion:**

These results suggest that PGN_0274 and PGN_1740 play a key role in biofilm formation by *P. gingivalis*.

**Electronic supplementary material:**

The online version of this article (doi:10.1186/1472-6831-15-4) contains supplementary material, which is available to authorized users.

## Background

The anaerobic Gram-negative bacterium *Porphyromonas gingivalis* is considered one of the important etiological agents of periodontal disease [1]. To colonize and survive in the gingival crevice, *P. gingivalis* must be capable of sensing and responding to the prevailing environmental conditions, including variations in temperature, oxygen tension, pH, nutrient availability and the presence of other bacterial cells. *P. gingivalis* possesses transcriptional regulators that have been implicated in protection against heat shock stress or oxidative stress, such as RprY [2, 3] and OxyR [4]. In addition, this bacterium and other bacteria form biofilms to protect against environmental stress [5]. Dental plaque, a multispecies biofilm, is organized on the tooth surface and periodontal tissues of the human oral cavity [6]. Oral bacteria in the biofilms survive in the gingival crevice for a long period of time, leading to gingivitis that can eventually progress into periodontitis. Understanding how bacteria escape environmental stress is very important for the prevention of periodontal disease.

Extracytoplasmic function (ECF) sigma factors serve as bacterial transcriptional regulators in the response to various stresses. The wild-type *P. gingivalis* 33277 genome encodes six ECF sigma factors (PGN_0274, PGN_0319, PGN_0450, PGN_0970, PGN_1108 and PGN_1740; GenBank: AP009380) [7]. PGN_1108 (W83 ORF number: PG1318) plays a role in the regulation of mutation frequency in the bacterium [8]. PGN_0274 (W83 ORF number: PG0162) and PGN_0450 (W83 ORF number: PG1660) may be involved in the post-transcriptional regulation of gingipain [9], and PGN_1740 (W83 ORF number: PG1827) is required for survival of the bacterium in the presence of oxygen and oxidative stress, hemin uptake and virulence [9, 10].

In this study, to analyze the role of ECF sigma factors in *P. gingivalis* biofilm formation, disruption of the ECF sigma factors, except PGN_1108, was performed. The PGN_1108-defective mutant may have a mutator phenotype, and we therefore excluded it from our experiments in this study [8]. The PGN_0274 and PGN_1740-defective mutants exhibited enhanced biofilm formation, but the complemented strains did not. These results suggest that the PGN_0274 and PGN_1740 ECF sigma factors are involved in the regulation of biofilm formation in the bacterium.

## Methods

### Bacterial strains and cell culture conditions

All bacterial strains and plasmids used in the present study are listed in Table 1. *P. gingivalis* cells were grown anaerobically (10% CO_2_, 10% H_2_ and 80% N_2_) in enriched brain heart infusion (BHI) broth and on enriched tryptic soy (TS) agar [11]. For blood agar plates, defibrinated laked sheep blood was added to enriched TS agar at 5%. For selection and maintenance of antibiotic-resistant *P. gingivalis* strains, the following antibiotics were added to the medium: 15 μg/ml erythromycin (Em), and 0.7 μg/ml tetracycline (Tc).

**Table 1:** **Bacterial strains and plasmids used in this study**

Strain or plasmid	Description	Reference or source
*E. coli* strain		
DH5α	General-purpose host strain for cloning	Invitrogen
*P. gingivalis* strain		
33277	wild type	ATCC
KDP314	PGN_0274*::ermF ermAM,* Em^r^	This study
KDP315	PGN_0319*::ermF ermAM,* Em^r^	This study
KDP316	PGN_0450*::ermF ermAM,* Em^r^	This study
KDP317	PGN_0970*::ermF ermAM,* Em^r^	This study
KDP319	PGN_1740*::ermF ermAM,* Em^r^	This study
KDP314C	KDP314/pKD828*,* Em^r^ Tc^r^	This study
KDP319C	KDP319/pKD829*,* Em^r^ Tc^r^	This study
*E. coli* plasmid		
pGEM-T Easy	Ap^r^, plasmid vector for TA cloning	Promega
pKD355	Ap^r^ *,* contains the *ermF ermAM* DNA cassette between *Eco*RI and *Bam*HI of pUC18	12
pKD814	Ap^r^, contains the 1.0-kb PCR-amplified fragment (PGN_0450 region) in pGEM-T Easy	This study
pKD817	Ap^r^, contains the 1.5-kb PCR-amplified fragment (PGN_0274 region) in pGEM-T Easy	This study
pKD818	Ap^r^, contains the 2.0-kb PCR-amplified fragment (PGN_0319 region) in pGEM-T Easy	This study
pKD819	Ap^r^, contains the 2.0-kb PCR-amplified fragment (PGN_0970 region) in pGEM-T Easy	This study
pKD821	Ap^r^, contains the 2.0-kb PCR-amplified fragment (PGN_1740 region) in pGEM-T Easy	This study
pKD822	Ap^r^ Em^r^, contains the *ermF ermAM* DNA cassette at *Bam*HI site within PGN_0274 of pKD817	This study
pKD823	Ap^r^ Em^r^, contains the *ermF ermAM* DNA cassette at *Bam*HI site within PGN_0319 of pKD818	This study
pKD824	Ap^r^ Em^r^, contains the *ermF ermAM* DNA cassette at *Bam*HI site within PGN_0450 of pKD814	This study
pKD825	Ap^r^ Em^r^, contains the *ermF ermAM* DNA cassette at *Bgl*II site within PGN_0970 of pKD819	This study
pKD827	Ap^r^ Em^r^, contains the *ermF ermAM* DNA cassette at *Bam*HI site within PGN_1740 of pKD821	This study
*P. gingivalis* plasmid		
pT-COW	Ap^r^ Tc^r^, *E. coli-P. gingivalis* shuttle plasmid	14
pKD828	Ap^r^ Tc^r^, pT-COW- PGN_0274^*+*^	This study
pKD829	Ap^r^ Tc^r^, pT-COW- PGN_1740^*+*^	This study

### Construction of ECF sigma factor mutants and complemented mutant strains

To disrupt the ECF sigma factor genes, PGN_0274-, PGN_0319-, PGN_0450-, PGN_0970- and PGN_1740-encoding genes were PCR-amplified from the chromosomal DNA of *P. gingivalis* 33277 using Takara Ex Taq (Takara Bio, Otsu, Japan) and the gene-specific primers listed in Table 2. The amplified areas were a DNA fragment containing part of the 5′ end of each ECF sigma factor gene and the upstream region of the ATG initiation codon, and a DNA fragment containing the 3′ end of each sigma factor gene and the downstream region of its stop codon. Both fragments were then ligated into the multiple cloning site of T-vector (pGEM-T Easy Vector, Promega, Tokyo, Japan). A *Bam*HI-*Sac*I fragment (*Bgl*II-*Sac*I fragment for PGN_0970) containing the 3′ end of each sigma factor gene was extracted from the resulting plasmid and ligated into the *Bam*HI-*Sac*I site (*Bgl*II-*Sac*I fragment for PGN_0970) of the plasmid containing the 5′ end of the corresponding ECF gene. The *ermF*-*ermAM* cassette of pKD355 [12] was inserted into the *Bam*HI site within PGN_0274 of pKD817, PGN_0319 of pKD818, PGN_0450 of pKD814 and PGN_1740 of pKD821, or the *Bgl*II site within PGN_0970 of pKD819 to yield pKD822, pKD823, pKD824, pKD827 and pKD825, respectively. These plasmids were linearized by *Not*I digestion and introduced into *P. gingivalis* 33277 cells by electroporation as described previously [13], resulting in KDP314 (PGN_0274::*ermF ermAM*), KDP315 (PGN_0319:: *ermF ermAM*), KDP316 (PGN_0450:: *ermF ermAM*), KDP317 (PGN_0970:: *ermF ermAM*) and KDP319 (PGN_1740:: *ermF ermAM*). Correct gene replacement of these strains, which had been generated by double crossover recombination events, was verified by PCR and Southern blot analysis (data not shown).

**Table 2:** **Primers used in this study**

Name	Nucleotide sequence (5′-3′)
PGN0274-U-F	TCGACAGTTGATTGCCGAT
PGN0274-U-R-*Bam*HI	G**GGATCC**CCATCGAAAGACTGCAATCTGG
PGN0274-D-F-*Bam*HI	G**GGATCC**CATGACGACGCCGCTCCTGTCGAAA
PGN0274-D-R	TGTGCAAAAAAGGAAACAGC
PGN0319-U-F	GCTGCCGCTCCTTCTTCAT
PGN0319-U-R-*Bam*HI	G**GGATCC**CAAAGGCAGATCGTCCGGTA
PGN0319-D-F-*Bam*HI	G**GGATCC**CCTCCGATCATGCCCCTA
PGN0319-D-R	TCAGGCTCTTGTACAGATGGA
PGN0450-U-F	GGGATGTGGAGAAAAAGGAA
PGN0450-D-R	ATGACCACGGACAGGAAGAT
PGN0970-U-F	ACCGGGAAATAATTCTCAAGC
PGN0970-U-R-*Bgl*II	A**AGATCT**TCCAAAGAGGTCGGATAAGGA
PGN0970-D-F- *Bgl*II	A**AGATCT**TAGGCTGCCGAGGTACAGGA
PGN0970-D-R	ACACAAGCTACAGCCCCGTA
PGN1740-U-F	GAGGATCTCCCTGCCAATAAT
PGN1740-U-R-*Bam*HI	G**GGATCC**CACCCAGCCTTTGAAGTTGACA
PGN1740-D-F-*Bam*HI	G**GGATCC**CGCTCACTGTCATGCGAAAT
PGN1740-D-R	CCAACGGCTATTTAGCATCC
PGN0274-COMP-U-F-*Pst*I	C**CTGCAG**GCTGCTACTGTCTCGGACGTG
PGN0274-COMP-D-R-*Bam*HI	G**GGATCC**CGTTTGTGTTTGAGGCTGCAT
PGN1740-COMP-U-F-*Bam*HI	CG**GGATCC**CGAGTGCGATATCGGGAATCAG
PGN1740-COMP-D-R-*Bam*HI	CG**GGATCC**CGAGTTGATACGGCTGCTATGC

Restriction sites incorporated into oligonucleotides for subcloning are bold.

For complementation of PGN_0274 and PGN_1740, the whole ECF sigma factor gene region with its upstream and downstream flanking regions (0.5 kb) was PCR-amplified from the chromosomal DNA using Takara Ex Taq with the upper and lower primers (Table 2). The amplified DNA fragments were ligated into the multiple cloning site of pGEM T-Easy vector. The *Sph*I-*Bam*HI fragment of PGN_0274 or the *Bam*HI fragment of PGN_1740 were extracted from the resulting plasmid and ligated into the *Sph*I-*Bam*HI or *Bam*HI site of pT-COW [14], which was kindly provided by Professor N. B. Shoemaker (University of Illinois at Urbana-Champaign, USA). The resulting plasmids, pKD828 and pKD829, were introduced into KDP314 or KDP319 by electroporation, resulting in KDP314C and KDP319C, respectively, after 7 d incubation on enriched TS agar containing 0.7 μg/ml tetracycline. The presence of pT-COW-derived plasmid was verified by PCR and restoration of the mRNA of the mutated gene was established by RT-PCR (data not shown).

### Evaluation of biofilm formation ability

Biofilm formation was examined by the modified protocol of Saito [15]. In brief, *P. gingivalis* cells were inoculated into BHI broth, and precultured anaerobically at 37°C for 2 d. Fully grown cultures of the *P. gingivalis* strains had turbidity adjusted to OD_660_ = 0.1 with fresh medium, and then 1.5-ml aliquots were inoculated into collagen type-I-coated 12-well flat-bottom microplates (IWAKI Glass Co., Funabashi, Japan) and cultured anaerobically at 37°C for 2 d. The culture medium was then removed from each well and 0.5 ml of 0.1% crystal violet solution was added. After 15 min, the wells were rinsed three times with PBS and air-dried. The crystal violet remaining in the biofilm was solubilized with 0.5 ml of 1% SDS and absorbance was measured at A_600_ using a microplate reader (Molecular Devices, Sunnyvale, CA, USA). Biofilm mass was determined by crystal violet staining and adjusted for growth (A_600_ units per OD_660_ unit).

### Statistical analysis

The one-way ANOVA Test/Dunnett’s Multiple Comparison Test was used to compare the differences between 33277 and ECF mutants using GraphPad Prism version 6.0 for Windows (GraphPad Software, Inc., La Jolla, CA, USA). Data were considered significant if *p* < 0.05.

## Results

### Growth and biofilm formation ability of the *P. gingivalis*ECF sigma factor mutants

**Figure 1:**
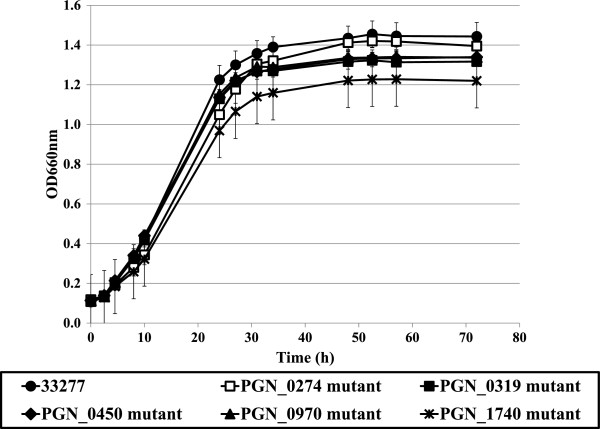
**Growth of** ***P. gingivalis*** **33277 and ECF sigma factor mutants.** Growth curves of *P. gingivalis* 33277 (wild-type; circle), PGN_0274 mutant (KDP314; open rectangle), PGN_0319 mutant (KDP315; closed rectangle), PGN_0450 mutant (KDP316; diamond), PGN_0970 mutant (KDP317; triangle), PGN_1740 mutant (KDP319; cross) in enriched BHI broth. The data shown are mean ± SD of triplicate experiments.

**Figure 2:**
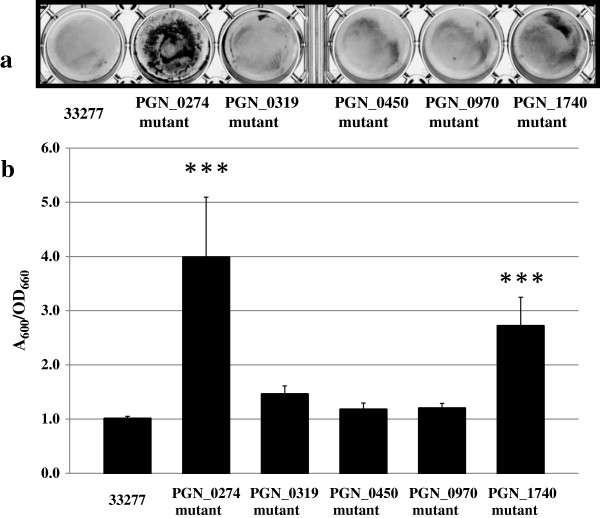
**Biofilm formation by homotypic** ***P. gingivalis*** **33277 or ECF sigma factor mutants.** The strains were grown on enriched BHI broth anaerobically at 37°C in collagen type-I-coated 12-well flat-bottom microplates. After 48 h of cultivation, the organized biofilm mass was evaluated by staining with crystal violet. **(a)** The photographs are a representative sample of each experimental strain. **(b)** Biofilm formation determined by crystal violet staining and adjusted for growth (A_600_ units per OD_660_ unit). The data shown are mean ± SD of triplicate experiments. ***, *p* < 0.001, by a one-way ANOVA Test/Dunnett’s Multiple Comparison Test.

All five ECF mutants grew more slowly than the wild-type strain in exponential phase, and final yields of the ECF mutants were less than that of the wild-type following a 48-h incubation under anaerobic conditions (Figure 1). The PGN_1740 mutant showed remarkably slow growth compared with wild-type and other ECF mutants. To evaluate the relationship between the biofilm formation activity and ECF sigma factors, the biofilm formation was examined for the wild-type and five ECF mutants. After crystal violet staining of the biofilm, it was first solubilized with ethanol. The crystal violet remaining in the wild-type, PGN_0319, PGN_0450, PGN_0970 and PGN_1740 mutant biofilm was solubilized and extracted, but in the PGN_0274 mutant, solubilization and extraction were not complete (see Additional file 1). Thus, the biofilm mass of all tested strains was dissolved with SDS and measured. Among the ECF mutants, the biofilm mass of the PGN_0274 and PGN_1740 mutants was higher than the wild-type (Figure 2). To confirm collagen type-I influences biofilm formation, we investigated the biofilm formation using a non-coated plate. The results were almost the same (see Additional file 2), as the PGN_0274 mutant produced more biofilm than the wild-type. However, the wild-type strain and the PGN_1740 mutant were not statistically different. This result suggested the biofilm formation by the PGN_1740 mutant was influenced by the environmental situation, such as the presence of collagen type-I.

### Complementation of the PGN_0274- and PGN_1740-defective mutants

**Figure 3:**
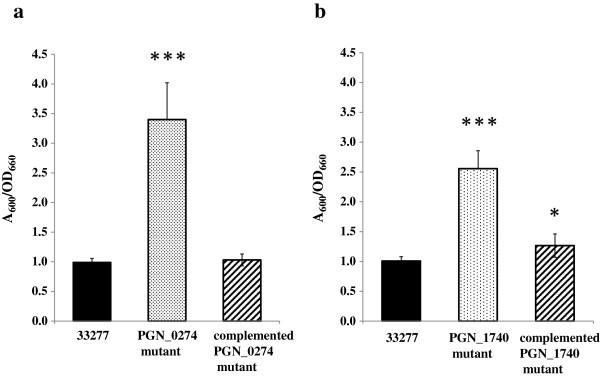
**Biofilm formation by homotypic** ***P. gingivalis*** **33277, ECF sigma factor mutant and complemented mutant strain.** The strains were grown on enriched BHI broth anaerobically at 37°C in collagen type-I-coated 12-well flat-bottom microplates. After 48 h of cultivation, the organized biofilm mass was evaluated by staining with crystal violet. **(a)** Biofilm formation of 33277, PGN_0274 mutant and the complemented mutant strain were compared. **(b)** Biofilm formation of 33277, PGN_1740 mutant and the complemented mutant strain were compared. Biofilm formation determined by crystal violet staining and adjusted for growth (A_600_ units per OD_660_ unit). The data shown are mean ± SD of triplicate experiments. *, *p* < 0.05, and ***, *p* < 0.001, by a one-way ANOVA Test/Dunnett’s Multiple Comparison Test.

To determine if the enhanced biofilm mass was caused by the deletion of PGN_0274 and PGN_1740, we constructed strains where the PGN_0274 and PGN_1740 were restored. The PGN_0274 and PGN_1740 complemented strains were constructed by introduction of the pT-COW containing the wild-type PGN_0274 and PGN_1740 into each of the mutants. This complementation restored the biofilm formation ability to the wild-type levels (Figure 3). These results support the concept that PGN_0274 and PGN_1740 play an important role in controlling *P. gingivalis* biofilm formation.

## Discussion

Bacteria sometimes encounter an environment unfavorable to their survival. The human oral microbiota is also often influenced by various stresses; hence, it must possess the ability to defend itself. Two principal regulatory mechanisms interact with cytoplasmic and extracytoplasmic regions via alternative ECF sigma factors and phosphorylation-dependent response regulators (two-component systems, TCSs) [16, 17]. ECF sigma factors have been shown to regulate cell envelope-related processes (involving maintenance of the membrane/periplasmic architecture), such as secretion, synthesis of exopolysaccharides, iron export and efflux synthesis of extracellular proteases [18]. Bacterial core RNA polymerase (composed of two α subunits, β subunit and β’ subunit) binds sigma factors. Multiple sigma factors are the bacterial transcription initiation factors that enable specific binding of RNA polymerase to gene promoters. In contrast, TCSs typically consist of a membrane-bound histidine kinase that senses a specific environmental stimulus and a corresponding response regulator that mediates the cellular response, mostly through differential expression of target genes [19]. Interestingly, a transcriptional regulator in *Methylobacterium extorquens*, PhyR, has been identified and determined to combine domains of both systems [20]. Taken together, ECF sigma factors and TCS are essential factors that protect bacteria from environmental stress.

Several *P. gingivalis* ECF sigma factors have been previously described. Nevertheless, there is no information on the ECF sigma factors that may operate in this bacterium in response to biofilm formation. In *Bacillus subtilis* and *Pseudomonas aeruginosa*, ECF sigma factors are involved in regulating biofilm development [21, 22]. In this study, we investigated whether biofilm formation of *P. gingivalis* is regulated by ECF sigma factors. This study demonstrated that PGN_0274 and PGN_1740 mutants yielded higher biofilm formation than that obtained with the wild-type or the other ECF sigma factor mutants. The inactivation of PGN_1740 also increased the expression of *fimS* at the transcriptional level [9]. Fimbriae and minor fimbriae influence monospecies biofilms [23]. The transcriptional level of *fimS* was examined using RT-PCR, which showed the *fimS* expression was downregulated (see Additional file 3). The results showed FimS may not be involved in controlling biofilm formation. Further work is needed to clarify this point.

The biofilm assay revealed that ethanol did not completely dissolve the biofilm mass and extract the crystal violet stain for the PGN_0274 mutant biofilm (see Additional file 1). Therefore, we dissolved the biofilm mass with SDS and measured the resulting crystal violet present in the sample. The need for a more stringent solvent suggested that the biofilm matrix around the mutant is composed partly of a protein component. The biofilm extracellular polymeric substances (EPS), composed of exopolysaccharides, proteins, nucleic acids and lipids, play a role as a defense structure, protecting bacteria from the host immune system and antimicrobial therapy [24]. Protein is a major component of EPS [25]. As the metabolic pathways of the PGN_0274 mutant are changed by the loss of the PGN_0274 ECF sigma factor, the protein yields in the PGN_0274 mutant are more abundant than those in the wild-type and the other mutants. Therefore, we examined the protein profile of the ECF sigma mutants compared with the wild-type (see Additional file 4). The degradation of 75-k–250-k Da proteins were demonstrated in the wild-type, PGN_0319, PGN_0450, PGN_0970 and PGN_1740 mutants, but not in the PGN_0274 mutant. This alteration was not observed in the presence of the proteinase inhibitors TLCK and leupeptin. The protein profiles of the wild-type and ECF sigma mutants were almost identical. Taken together, these results suggest this apparent difference in solubility might be explained by the decrease of Kgp and Rgp activity in the PGN_0274 mutant [9]. Kgp suppresses biofilm formation and Rgp controls microcolony morphology [26]. The *sinR* ortholog PGN_0088, a transcriptional regulator, acts as a negative regulator of exopolysaccharide accumulation in wild-type *P. gingivalis* [27]. PGN_0274 may control different metabolic pathways than PGN_0088 and act as a negative regulator of protein accumulation.

In conclusion, we have identified that PGN_0274 and PGN_1740 play a key role in *P. gingivalis* biofilm formation. These results show for the first time that *P. gingivalis* ECF sigma factors are involved in biofilm formation. PGN_0274 is involved in the post-transcriptional regulation of gingipains [8]. Gingipain is a very important virulence factor in *P. gingivalis*, because gingipains destroy periodontal tissue, immunoglobulins and complement factors [28, 29]. As PGN_1740 plays a significant role in oxidative stress responses in the bacterium [8, 9], the survival of the PGN_1740 mutant was reduced in the presence of host cells [9]. We also observed this in Ca9-22 cells (data not shown). Taken together, these results show that ECF sigma factors PGN_0274 and PGN_1740 are involved in the virulence of *P. gingivalis*. Further studies on the roles of the *P. gingivalis* ECF sigma factors, PGN_0274 and PGN_1740, will help us understand the ability of *P. gingivalis* to colonize and survive in the gingival crevice, and therefore act as a human pathogen.

## Conclusions

The mass of the biofilm of the PGN_0274 and PGN_1740 mutants was higher than that in the wild-type, suggesting *P. gingivalis* extracytoplasmic function sigma factors PGN_0274 and PGN_1740 are involved in biofilm formation.

## Electronic supplementary material

Additional file 1: **Comparisons of biofilm treated with ethanol or SDS.** (PPTX 456 KB)

Additional file 2: **Biofilm formation by homotypic** ***P. gingivalis*** **33277 or ECF sigma factor mutants using non-coated microplate.** (PPTX 80 KB)

Additional file 3: **The RNA expression of** ***fimS*** **in** ***P. gingivalis*** **33277, PGN**_**1740 mutant and complemented mutant strain.** (PPTX 101 KB)

Additional file 4: **Protein profile on an SDS-PAGE gel.** (PPTX 343 KB)

## Abbreviations

ECF: Extracytoplasmic function
BHI: Brain heart infusion
TS: Tryptic soy
Em: Erythromycin
Tc: Tetracycline
PCR: Polymerase chain reaction
ANOVA: Analysis of variance
TCS: Two-component systems
SDS: Sodium dodecyl sulfate
EPS: Extracellular polymeric substances.

## References

[1] Holt SC, Ebersole JL (2005). *Porphyromonas gingivalis*, *Treponema denticola*, and *Tannerella forsythia*: the “red complex”, a prototype polybacterial pathogenic consortium in periodontitis. Periodontol 2000.

[2] Duran-Pinedo AE, Nishikawa K, Duncan MJ (2007). The RprY response regulator of *Porphyromonas gingivalis*. Mol Microbiol.

[3] Krishnan K, Duncan MJ (2013). Role of sodium in the RprY-dependent stress response in *Porphyromonas gingivalis*. PLoS One.

[4] Diaz PI, Slakeski N, Reynolds EC, Morona R, Rogers AH, Kolenbrander PE (2006). Role of *oxyR* in the oral anaerobe *Porphyromonas gingivalis*. J Bacteriol.

[5] Socransky SS, Haffajee AD (2002). Dental biofilms: difficult therapeutic targets. Periodontol 2000.

[6] Kolenbrander PE (2000). Oral microbial communities: biofilms, interactions, and genetic systems. Annu Rev Microbiol.

[7] Naito M, Hirakawa H, Yamashita A, Ohara N, Shoji M, Yukitake H (2008). Determination of the genome sequence of *Porphyromonas gingivalis* strain ATCC 33277 and genomic comparison with strain W83 revealed extensive genome rearrangements in *P. gingivalis*. DNA Res.

[8] Kikuchi Y, Ohara N, Ueda O, Hirai K, Shibata Y, Nakayama K (2009). *Porphyromonas gingivalis* mutant defective in a putative extracytoplasmic function sigma factor shows a mutator phenotype. Oral Microbiol Immunol.

[9] Dou Y, Osbourne D, McKenzie R, Fletcher HM (2010). Involvement of extracytoplasmic function sigma factors in virulence regulation in *Porphyromonas gingivalis* W83. FEMS Microbiol Lett.

[10] Yanamandra SS, Sarrafee SS, Anaya-Bergman C, Jones K, Lewis JP (2012). Role of the *Porphyromonas gingivalis* extracytoplasmic function sigma factor, SigH. Mol Oral Microbiol.

[11] Nakayama K, Kadowaki T, Okamoto K, Yamamoto K (1995). Construction and characterization of arginine-specific cysteine proteinase (Arg-gingipain)-deficient mutants of *Porphyromonas gingivalis*. Evidence for significant contribution of Arg-gingipain to virulence. J Biol Chem.

[12] Ueshima J, Shoji M, Ratnayake DB, Abe K, Yoshida S, Yamamoto K (2003). Purification, gene cloning, gene expression, and mutants of Dps from the obligate anaerobe *Porphyromonas gingivalis*. Infect Immun.

[13] Nakayama K (1994). Rapid viability loss on exposure to air in a superoxide dismutase-deficient mutant of *Porphyromonas gingivalis*. J Bacteriol.

[14] Gardner RG, Russell JB, Wilson DB, Wang GR, Shoemaker NB (1996). Use of a modified Bacteroides-Prevotella shuttle vector to transfer a reconstructed β-1,4-D-endoglucanase gene into *Bacteroides uniformis* and *Prevotella ruminicola* B_1_4. Appl Environ Microbiol.

[15] Saito Y, Fujii R, Nakagawa KI, Kuramitsu HK, Okuda K, Ishihara K (2008). Stimulation of *Fusobacterium nucleatum* biofilm formation by *Porphyromonas gingivalis*. Oral Microbiol Immunol.

[16] Raivio TL (2005). Envelope stress responses and Gram-negative bacterial pathogenesis. Mol Microbiol.

[17] Jordan S, Hutchings MI, Mascher T (2008). Cell envelope stress response in Gram-positive bacteria. FEMS Microbiol Rev.

[18] Bashyam MD, Hasnain SE (2004). The extracytoplasmic function sigma factors: role in bacterial pathogenesis. Infect Genet Evol.

[19] Mascher T, Helmann JD, Unden G (2006). Stimulus perception in bacterial signal-transducing histidine kinases. Microbiol Mol Biol Rev.

[20] Francez-Charlot A, Frunzke J, Reichen C, Ebneter JZ, Gourion B, Vorholt JA (2009). Sigma factor mimicry involved in regulation of general stress response. Proc Natl Acad Sci USA.

[21] Bordi C, de Bentzmann S (2011). Hacking into bacterial biofilms: a new therapeutic challenge. Ann Intensive Care.

[22] Luo Y, Asai K, Sadaie Y, Helmann JD (2010). Transcriptomic and phenotypic characterization of a *Bacillus subtilis* strain without extracytoplasmic function sigma factors. J Bacteriol.

[23] Lin X, Wu J, Xie H (2006). *Porphyromonas gingivalis* minor fimbriae are required for cell-cell interactions. Infect Immun.

[24] Flemming HC, Wingender J (2010). The biofilm matrix. Nat Rev Microbiol.

[25] Ali Mohammed MM, Nerland AH, Al-Haroni M, Bakken V (2013). Characterization of extracellular polymeric matrix, and treatment of *Fusobacterium nucleatum* and *Porphyromonas gingivalis* biofilms with DNase I and proteinase K. J Oral Microbiol.

[26] Kuboniwa M, Amano A, Hashino E, Yamamoto Y, Inaba H, Hamada N (2009). Distinct roles of long/short fimbriae and gingipains in homotypic biofilm development by *Porphyromonas gingivalis*. BMC Microbiol.

[27] Yamamoto R, Noiri Y, Yamaguchi M, Asahi Y, Maezono H, Kuboniwa M (2013). The *sinR* ortholog PGN_0088 encodes a transcriptional regulator that inhibits polysaccharide synthesis in *Porphyromonas gingivalis* ATCC 33277 biofilms. PLoS One.

[28] Holt SC, Kesavalu L, Walker S, Genco CA (1999). Virulence factors of *Porphyromonas gingivalis*. Periodontol 2000.

[29] Lamont RJ, Jenkinson HF (1998). Life below the gum line: pathogenic mechanisms of *Porphyromonas gingivalis*. Microbiol Mol Biol Rev.

[CR30] The pre-publication history for this paper can be accessed here:http://www.biomedcentral.com/1472-6831/15/4/prepub

