# Supplementary material for: Role of extracytoplasmic function sigma factors in biofilm formation of Porphyromonas gingivalis
Source: BMC Oral Health. 2015 Jan 17;15:4. doi: 10.1186/1472-6831-15-4 (PMC4324044; doi:10.1186/1472-6831-15-4)
Supplement: Supplementary file 1 — Additional file 1: Comparisons of biofilm treated with ethanol or SDS. (PPTX 456 KB) [file 12903_2014_492_MOESM1_ESM.pptx]

## Slide 1
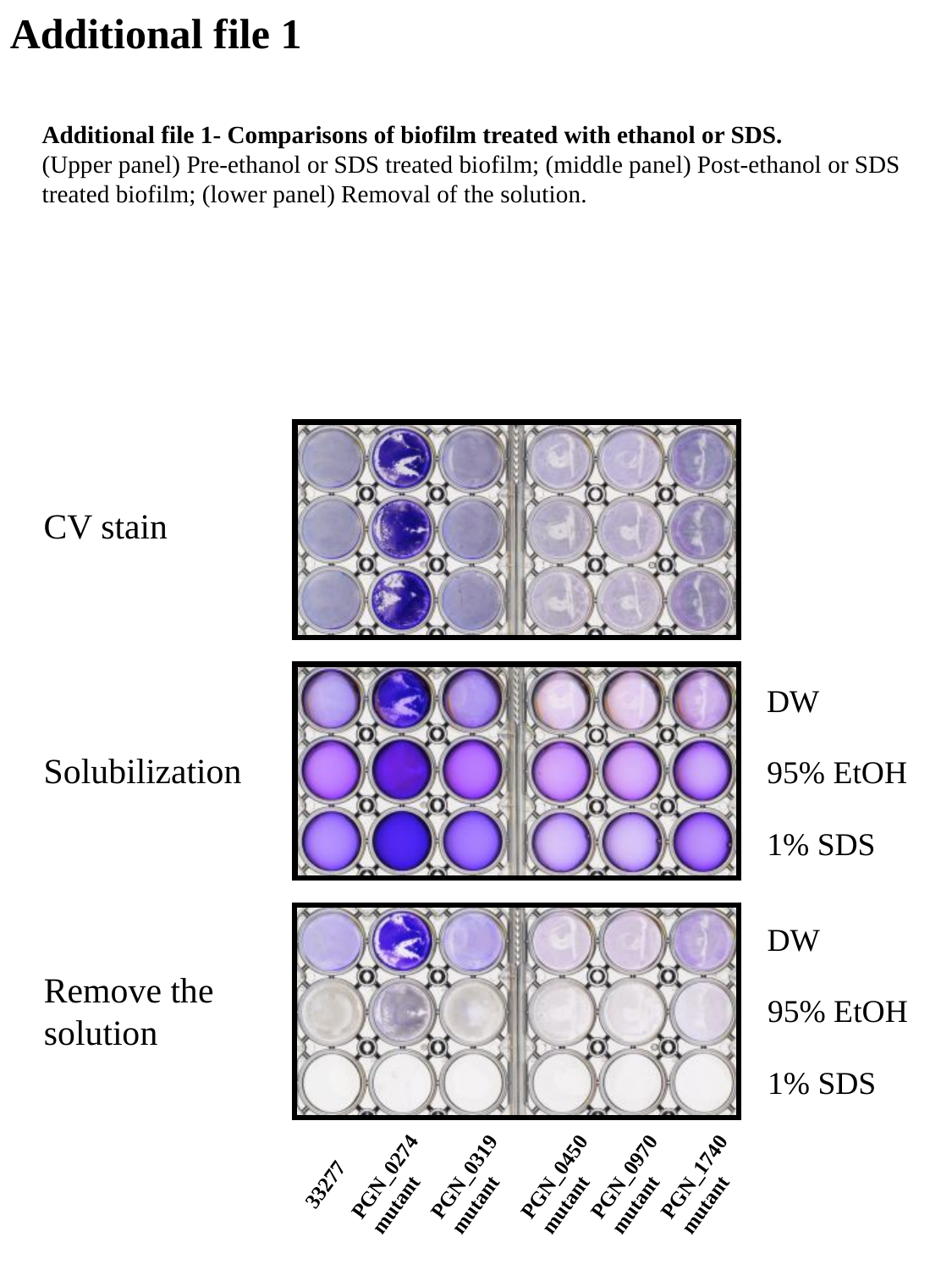

Additional file 1
Additional file 1- Comparisons of biofilm treated with ethanol or SDS.
(Upper panel) Pre-ethanol or SDS treated biofilm; (middle panel) Post-ethanol or SDS
treated biofilm; (lower panel) Removal of the solution.
CV stain
DW
95% EtOH
1% SDS
Solubilization
DW
95% EtOH
1% SDS
Remove the
solution
PGN_0274
mutant
PGN_0319
mutant
PGN_0450
mutant
PGN_0970
mutant
PGN_1740
mutant
33277
