# Supplementary material for: Role of extracytoplasmic function sigma factors in biofilm formation of Porphyromonas gingivalis
Source: BMC Oral Health. 2015 Jan 17;15:4. doi: 10.1186/1472-6831-15-4 (PMC4324044; doi:10.1186/1472-6831-15-4)
Supplement: Supplementary file 2 — Additional file 2: Biofilm formation by homotypic P. gingivalis 33277 or ECF sigma factor mutants using non-coated microplate. (PPTX 80 KB) [file 12903_2014_492_MOESM2_ESM.pptx]

## Slide 1
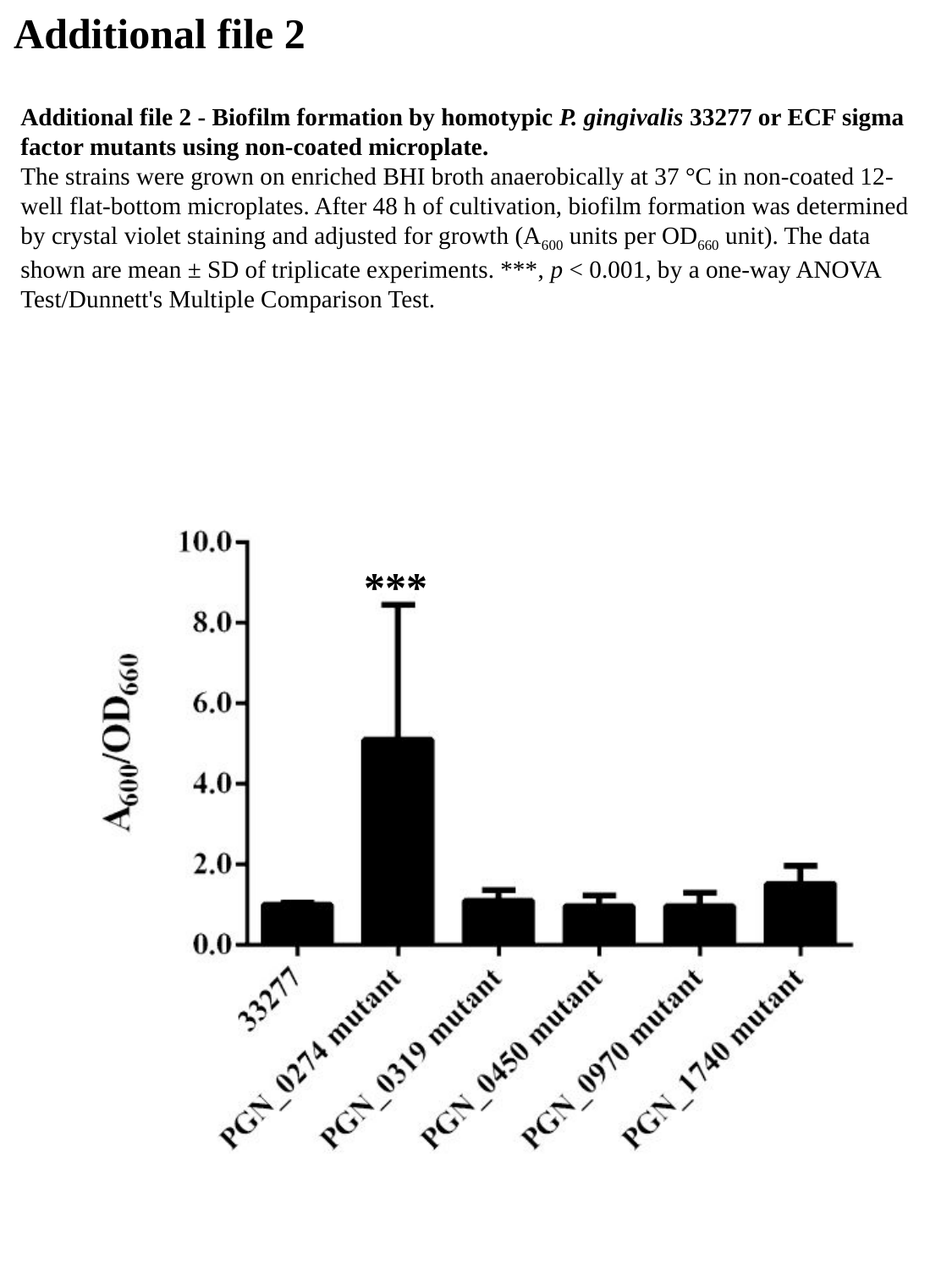

Additional file 2
Additional file 2 - Biofilm formation by homotypic P. gingivalis 33277 or ECF sigma
factor mutants using non-coated microplate.
The strains were grown on enriched BHI broth anaerobically at 37 °C in non-coated 12-well flat-bottom microplates. After 48 h of cultivation, biofilm formation was determined by crystal violet staining and adjusted for growth (A600 units per OD660 unit). The data shown are mean ± SD of triplicate experiments. ***, p < 0.001, by a one-way ANOVA Test/Dunnett's Multiple Comparison Test.
***
