# Supplementary material for: Role of extracytoplasmic function sigma factors in biofilm formation of Porphyromonas gingivalis
Source: BMC Oral Health. 2015 Jan 17;15:4. doi: 10.1186/1472-6831-15-4 (PMC4324044; doi:10.1186/1472-6831-15-4)
Supplement: Supplementary file 3 — Additional file 3: The RNA expression of fimS in P. gingivalis 33277, PGN_1740 mutant and complemented mutant strain. (PPTX 101 KB) [file 12903_2014_492_MOESM3_ESM.pptx]

## Slide 1
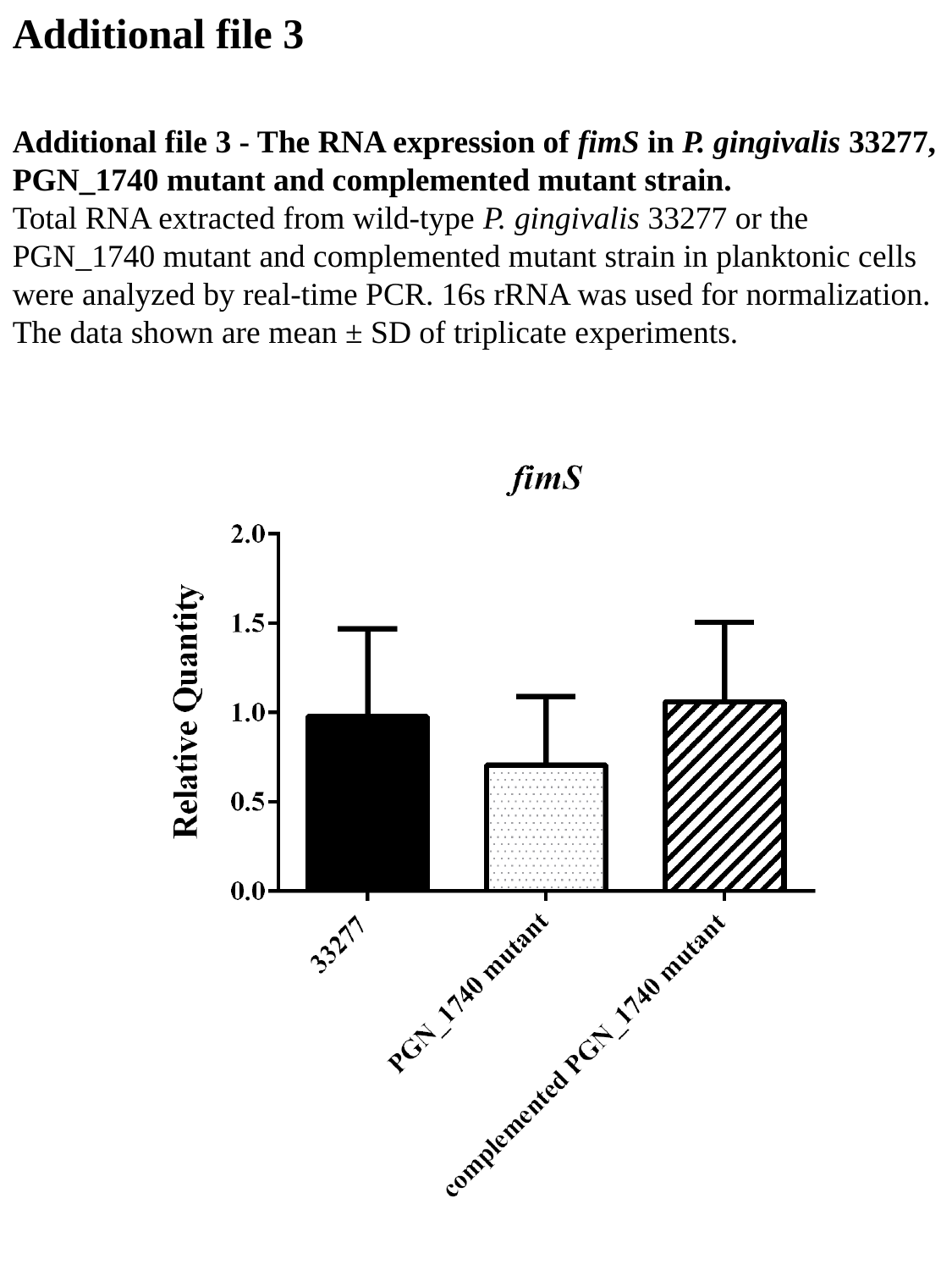

Additional file 3
Additional file 3 - The RNA expression of fimS in P. gingivalis 33277, PGN_1740 mutant and complemented mutant strain.
Total RNA extracted from wild-type P. gingivalis 33277 or the PGN_1740 mutant and complemented mutant strain in planktonic cells were analyzed by real-time PCR. 16s rRNA was used for normalization. The data shown are mean ± SD of triplicate experiments.
