# Supplementary material for: Role of extracytoplasmic function sigma factors in biofilm formation of Porphyromonas gingivalis
Source: BMC Oral Health. 2015 Jan 17;15:4. doi: 10.1186/1472-6831-15-4 (PMC4324044; doi:10.1186/1472-6831-15-4)
Supplement: Supplementary file 4 — Additional file 4: Protein profile on an SDS-PAGE gel. (PPTX 343 KB) [file 12903_2014_492_MOESM4_ESM.pptx]

## Slide 1
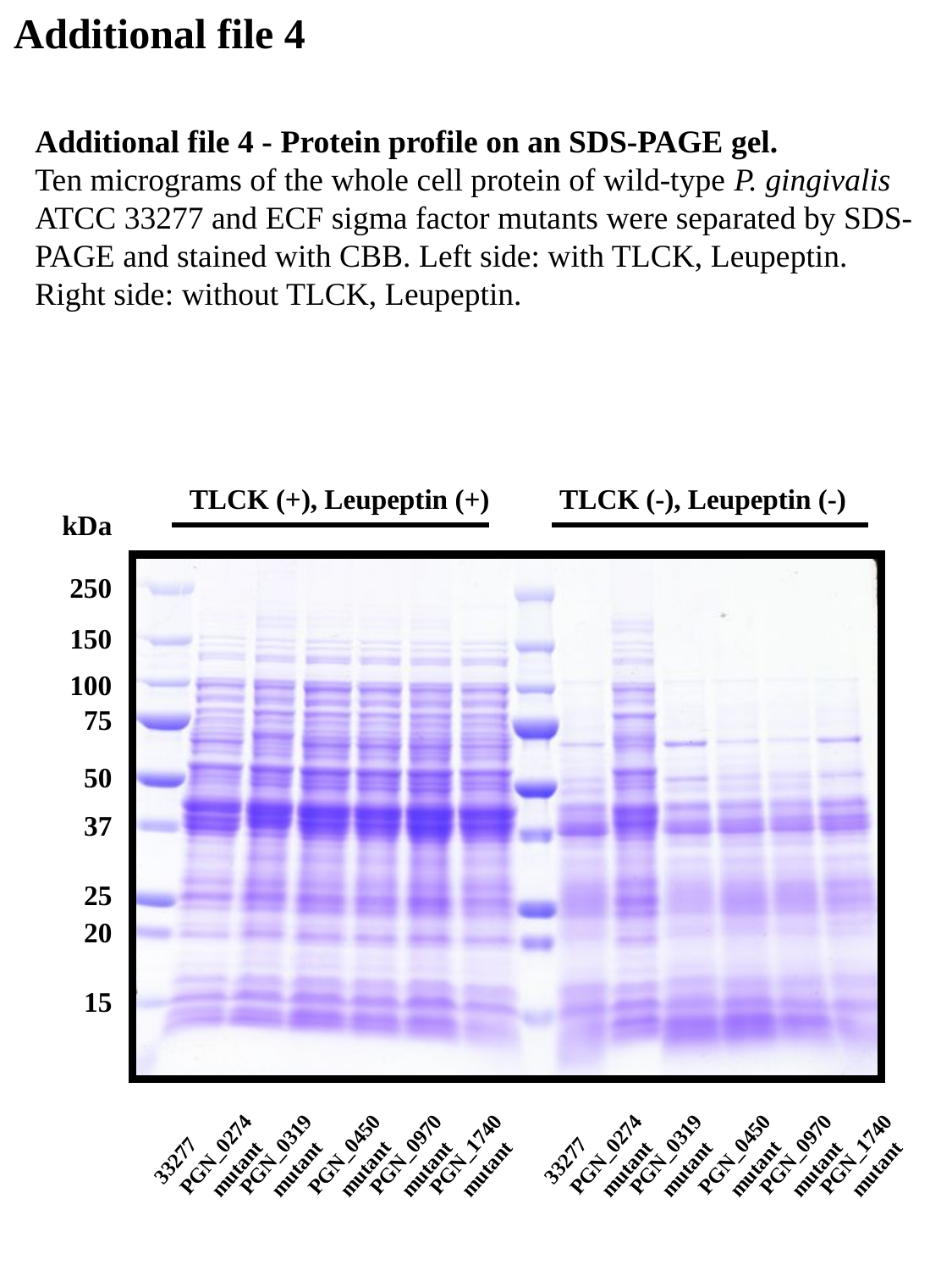

Additional file 4
Additional file 4 - Protein profile on an SDS-PAGE gel.
Ten micrograms of the whole cell protein of wild-type P. gingivalis ATCC 33277 and ECF sigma factor mutants were separated by SDS-PAGE and stained with CBB. Left side: with TLCK, Leupeptin. Right side: without TLCK, Leupeptin.
TLCK (+), Leupeptin (+)
TLCK (-), Leupeptin (-)
kDa
250
150
100
75
50
37
25
20
15
PGN_0274
mutant
PGN_0319
mutant
PGN_0450
mutant
PGN_0970
mutant
PGN_1740
mutant
PGN_0274
mutant
PGN_0319
mutant
PGN_0450
mutant
PGN_0970
mutant
PGN_1740
mutant
33277
33277
